# Whānau Māori and Pacific peoples’ knowledge, perceptions, expectations and solutions regarding antibiotic treatment of upper respiratory tract infections: a qualitative study

**DOI:** 10.1186/s12879-023-08431-5

**Published:** 2023-07-10

**Authors:** S Thaggard, S Reid, AHY Chan, C White, L Fraser, BA Arroll, E Best, R Whittaker, S Wells, MG Thomas, SR Ritchie

**Affiliations:** 1 Nursing Department, Auckland University of Technology, Auckland, New Zealand; 2 Health Literacy NZ, Auckland, New Zealand; 3 Faculty of Medical and Health Sciences, University of Auckland, Auckland, New Zealand; 4 Turuki Health Care, Auckland, New Zealand; 5 502-301E Infection and Immunity, School of Medical Sciences, Faculty of Medical and Health Sciences, University of Auckland, Auckland, Private Bag 92019 New Zealand

## Abstract

**Introduction:**

The rate of community antibiotic use is high in Aotearoa New Zealand (NZ) when compared to other nations, and in NZ, as in most other nations, antibiotics are very commonly prescribed for self-limiting upper respiratory tract infections (URTIs). Resources that build knowledge, perceptions and understanding can potentially reduce unnecessary antibiotic consumption.

**Methods:**

To inform the content of educational resources, we conducted an in-depth qualitative study with 47 participants via 6 focus groups of the knowledge, attitudes, and expectations of whānau Māori and Pacific peoples about antibiotics and URTIs.

**Results:**

Focus groups with 47 participants identified four themes: Knowledge that might influence expectations to receive antibiotics for URTIs; Perceptions - the factors that influence when and why to seek medical care for URTI; Expectations - the features of successful medical care for URTI; Solutions - how to build community knowledge about URTI and their treatment and prevention. Knowledge that might reduce expectations to receive antibiotics for URTI included confidence in the use of alternative remedies, knowledge that URTI are usually caused by viruses, and concerns about antibiotic adverse effects. Participants commonly reported that they would confidently accept their doctor’s recommendation that an antibiotic was not necessary for an URTI, provided that a thorough assessment had been performed and that treatment decisions were clearly communicated.

**Conclusion:**

These findings suggest that building patients’ knowledge and skills about when antibiotics are necessary, and increasing doctors’ confidence and willingness not to prescribe an antibiotic for patients with an URTI, could significantly reduce inappropriate antibiotic prescribing in NZ.

## Introduction

Antibiotic use in Aotearoa New Zealand (NZ) is the fifth highest among high income countries [1]. When compared with other developed nations, the amount of antibiotic dispensed by community pharmacies is very high in NZ, while the amount dispensed for hospital inpatients is relatively low [2]. Overall, medicines dispensed by community retail pharmacies comprise approximately 95% of all antibiotics dispensed for human use in NZ. Therefore, greater benefits can be achieved by reducing inappropriate antibiotic use in the community than by reducing inappropriate antibiotic consumption in hospitals [3, 4].

The incidence of some infectious diseases is higher in NZ than in many other countries, and this higher incidence of infectious diseases is especially marked in whānau (family) Māori (indigenous people of New Zealand) and Pacific peoples (people with Pacific Island ethnicity) [5]. The incidence of rheumatic fever provides the most striking example of ethnic disparity. The mean annual incidence of first episodes of hospitalisation for rheumatic fever in Auckland NZ, during 2014 to 2016 was 98.3/1000 person years for Pacific peoples, 35.5/1000 for Māori, 0.4/1000 for people of European or Other ethnicities, and 0.0/1000 for people of Asian ethnicities [6]. The reason for the marked ethnic disparities in the incidence of infectious diseases in NZ is likely to be multifactorial, including inter-generational socio-economic deprivation arising from colonisation, adverse experiences during health system interactions, and barriers to access to healthcare imposed by resource constraints. However, these gaps are modifiable as evidenced by a successful rheumatic fever prevention campaign that focussed on antibiotic treatment of sore throats for Māori and Pacific children [7].

Most of the antibiotics dispensed in NZ are thought to be prescribed for self-limiting viral upper respiratory tract infections (URTIs) [8]. Despite ethnic disparities in the incidence of infectious diseases in NZ, the per capita rate of antibiotic dispensing by community pharmacies in NZ appears to be broadly comparable for all ethnic groups [9]. In particular, all ethnic groups have a similar increase in antibiotic dispensing during the winter months, when viral URTIs are more prevalent [9].

Low levels of knowledge and information about antibiotics are likely to contribute to expectations to be prescribed antibiotics for URTIs [10, 11]. An important consequence of infectious disease disparities in NZ is that the potential benefits of improving knowledge about infectious diseases may be greater for whānau Māori and Pacific peoples than for people of other ethnicities. A qualitative study of 30 Māori found gaps in knowledge and information, and misconceptions about antibiotics, that had the potential to influence antibiotic use [12]. We therefore sought to expand this knowledge base to explore whānau Māori and Pacific people’s knowledge, perceptions, and expectations regarding antibiotic treatment of URTIs with the aim of informing development of educational resources that could build knowledge and skills and reduce the inappropriate prescribing of antibiotics.

## Methods

### Participants

Potential participants for focus groups were recruited through established community networks by members of the research team at Health Literacy NZ (a consultancy that seeks to improve system and service design for better, equitable health outcomes) and at a primary care practice. A purposive sampling approach was used to determine the inclusion and exclusion criteria for participants [13]. People who self-identified as Māori or Pacific people and were aged between 18 and 65 years old were eligible for inclusion. Participants were also required to be fluent in English as we wanted to ensure that there was a common language that could be used by all focus group participants and facilitators (to increase accuracy of data collection). Written informed consent was obtained from all participants prior to the focus groups. Ethical approval was provided by the Auckland Health Research Ethics Committee (AH21565).

### Focus group discussions

All methods were carried out in accordance with relevant guidelines and regulations including following guidelines from the EQUATOR network for reporting of qualitative research, the Declaration of Helsinki and the National Ethical Standards for Health and Disability Research. In addition, the research approach incorporated the four principles identified by Sir Mason Durie that underpin learning and research at the interface of indigenous knowledge and science: mutual respect, shared benefits, human dignity, and discovery. A semi-structured interview guide was used to shape and encourage the flow of dialogue. Focus groups were conducted using the Hui process for Māori participants and using the Talanoa method for Pacific participants. Focus group interviews were conducted by an experienced academic Pasifika registered nurse (ST), Māori health literacy experts experienced in leading focus groups (SR, CW), an Asian pharmacist with expertise in qualitative research and behaviour change (AC), and a Pakeha (New Zealander of European descent) Infectious Disease specialist (MT) in attendance to answer questions that arose.

The four essential components of the Hui process are: mihimihi (initial greeting), whakawhanaungatanga (making a connection/building relationships), kaupapa (attending to the purpose of the encounter) and poroporoaki/whakamutunga (closing the session) [14].

The Talanoa method reflects the cultural context, and is a trustworthy approach that helps Pacific people feel more involved and empowered [15, 16]. The Talanoa method is transferable across different Pacific groups and allows for a free flow of information in a focus group interview and flexibility to cross-validate answers through open ended questions between and amongst participants.

The aims and objectives were explained to each focus group prior to the interviews. A trained health professional (nurse, pharmacist, or infectious disease specialist) from the research team was present throughout each focus group. An important part of both the Hui process and the Talanoa method was that participants had the opportunity to ask questions, which were either answered when raised, or answered at the end of the focus group. Each focus group was recorded, and the recordings were transcribed by an independent transcriptionist, who signed a confidentiality agreement. Members of the research team also made written notes during each of the focus groups. Kai (light refreshments) and a koha (gift) in the form of a supermarket voucher were provided to all participants as a gesture of reciprocity for the sharing of knowledge and participation in the focus groups.

### Interview guide

An interview guide (supplementary material) that drew upon knowledge obtained from past surveys and published literature was used with each focus group to ensure consistency between the focus groups about the topics discussed. Participants were specifically asked about their knowledge of antibiotics, their perceptions of antibiotic treatment of URTIs, and their expectations in relation to being prescribed an antibiotic if either they or their child had an URTI. Participants were also asked for advice about how to improve public knowledge about why antibiotics should not be used for URTIs.

### Analysis

Analysis was undertaken using inductive and data driven thematic analytic process informed by naturalistic inquiry [17]. This method of analysis provides an “accessible and theoretically flexible approach” following a step-by-step process [18, 19]. The thematic analysis identified patterns within and across the interview data in relation to the participants’ experiences, views and perspectives. Provisional themes were identified and refined from transcribed data by the research team with discussion until consensus was attained.

## Results

A total of 47 participants: 21 Māori and 26 Pacific (19 Samoan, 4 Tongan, 2 Cook Islanders, 1 Kiribati) participants, comprising 43 women and 4 men, took part in six focus groups. Five of these were held in Auckland (NZ’s largest city, population 1.7 million) with 29 women and 4 men, and one held in Taranaki (regional city, population 120,000) with 13 Māori women and one Pacific woman. Each focus group lasted between 60 and 90 min. Thematic saturation was reached after the six focus groups.

Overall four major themes were identified: Knowledge that might influence expectations to receive antibiotics for URTIs, Perceptions - the factors that influence when and why to seek medical care for URTI, Expectations - the features of successful medical care for URTI, and Solutions - how to build community knowledge about URTI and their treatment and prevention.

### Theme 1: knowledge that might influence expectations to receive antibiotics for URTIs

**Fig. 1:**
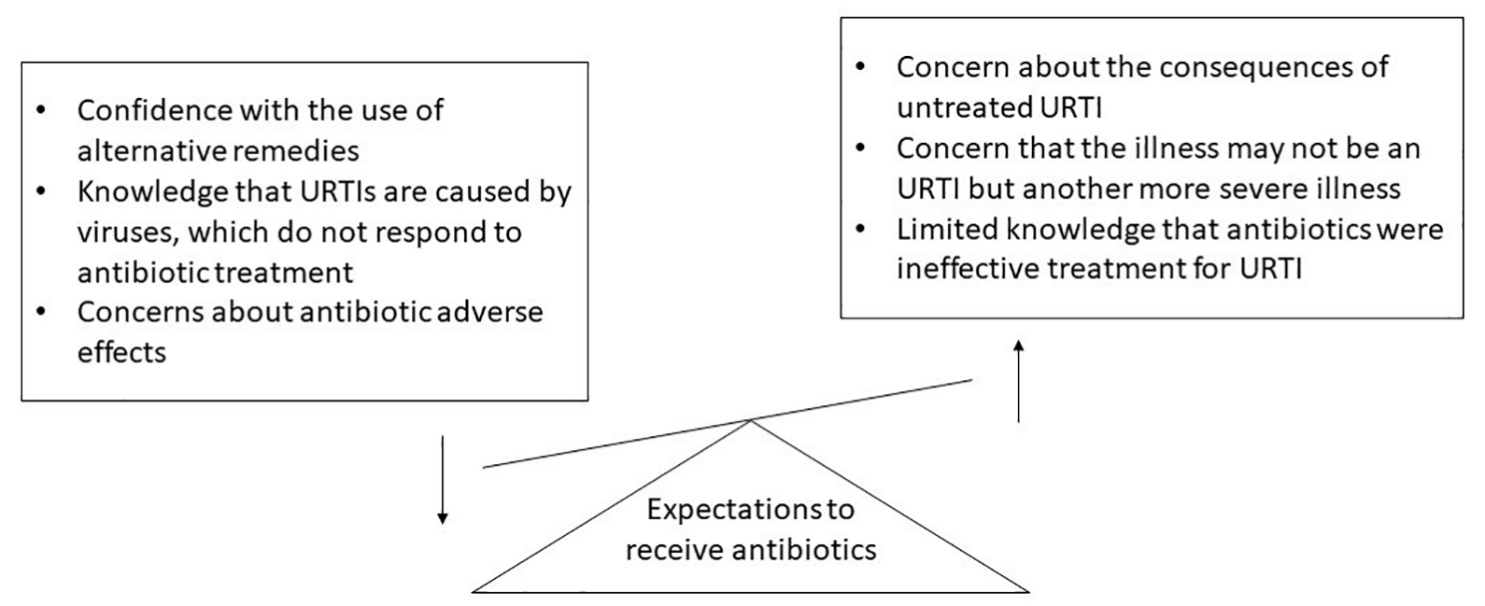
Theme 1: Participants’ knowledge that might influence expectations to receive antibiotics for URTIs

Focus group participants identified six factors that might either reduce or increase expectations to receive antibiotics for viral URTIs (Fig. 1).

(a) Confidence with the use of alternative remedies for URTIs.

Most participants stated that home remedies were useful for URTIs, either used alone or to supplement antibiotic treatment. Several participants described using home remedies for themselves or their children and only visiting their doctor if they or their children didn’t get better. The home remedies suggested by participants included: Vicks® rub, lemon and honey drinks, steam inhalations, garlic, rongoā Māori (traditional Māori healing including herbal remedies, physical remedies such as mirimiri (massage) and spiritual healing) and guava leaves.

P35: I’m so much more excited about the natural stuff than the antibiotics.

P12: I do a lemon drink and honey. I just make my kids lemon drink and honey and that’s it. And I make them drink as much fluids as they can, otherwise, it’s Rongoā Māori.

P32: And kumarahou (a plant native to NZ that is used in the making of traditional herbal remedies). You put it in the tea and you drink it. It helps you get rid of the flu. You boil it. It’s a plant.

(b) Knowledge that URTIs are caused by viruses that do not respond to antibiotic treatment.

When participants were asked if antibiotics were used to treat viral conditions such as COVID-19, a few participants were quickly able to identify that COVID-19 was caused by a virus, apparent from the name coronavirus, and so antibiotics would not work for viral respiratory conditions such as COVID-19.

P7: You don’t need antibiotics, you’ve got a virus.

P19M: No, I don’t think so. I don’t know. ‘Cause it’s viral, it’s not bacterial.

(c) Concerns about antibiotics adverse effects.

Most participants knew that antibiotic treatment could result in a range of adverse effects, with several reporting that they had suffered from one or more adverse effects.

P16: I think you have to be careful with what you prescribe people in terms of various antibiotics. Because some people might have allergic reactions to specific types.

P17: (talking about an experience related to a prescribed antibiotic) Yes, my son he had ulcers in his mouth. They spread around when he was given it, I don’t know what it was. Then we came back in because it just got worse and then he said he was allergic to it.

Some participants knew that use of antibiotics could result in the infecting bacteria becoming resistant to the antibiotic. However, this knowledge was commonly expressed as antibiotics having an effect on the immune system.

P35: There’s some antibiotics that cure some things but may not cure other things, like MRSA. They can be given antibiotics and it does nothing for their infection until they get the right one. They become resistant.

P17: You can also become immune to it if you take it when you don’t need it or when you take it too much.

P30: I feel like in the end it’s going to give your body a weaker immune system.

(d) Concern about the consequences of untreated URTI.

Most participants reported that they were more likely to seek medical assessment, and consideration of antibiotic treatment, for vulnerable individuals such as young children or older adults with URTI that hadn’t responded to rest, fluids and home remedies, and for adults with a chronic condition such as asthma or diabetes. If there were concerns that the URTI has lasted longer than usual or been more severe than usual or that the illness may not be an URTI but another more severe illness such as rheumatic fever, then participants were more inclined to seek review and potential antibiotic treatment. Most participants in the Auckland focus groups were aware that they should seek medical assessment for young people with a sore throat, because of the risk that an untreated infection might lead to rheumatic fever.

P37: Sometimes there’s other infections that has the same symptoms, but you think it’s the same thing, but it could be something worse.

P32. I always take him when he’s got a sore throat. Only because we’ve got a family history of rheumatic fever. You can never be too sure.

(e) Limited knowledge that antibiotics were ineffective treatment for URTI.

In the early stages of each focus group’s discussion, only a few participants said that antibiotics kill bacteria, and that this process helps recovery from an infectious disease.

P14M: I’ve heard that they’re good at fighting bacteria. And it reduces infection. I pretty much get better every single time I take antibiotics.

P16: It’s supposed to kill off the bacteria.

Some participants were unsure about which medicines were antibiotics and asked whether the term “antibiotics” included antihistamines and anti-inflammatories as these were all used to treat swelling and inflammation. Some participants considered antibiotics might be indicated for some non-infectious diseases. One participant asked if she could take antibiotics for high blood pressure. Another participant thought that antibiotics were provided for gout. This discussion raised questions from some participants.

P14M: What’s the difference between antibiotics and antihistamines?

P39: Can you have an infection in the blood? Like if you have a toothache and then sometimes it travels to your blood. You need anti-virals for those.

P43: I thought Allopurinol would have been an antibiotic?

Most participants were not aware that antibiotics were not an effective treatment for URTI. A few participants knew that URTI were primarily caused by viruses. These people also knew antibiotics should not be used for these conditions. Some participants knew that antibiotics were seldom prescribed for “colds”.

P36 (talking about colds) It’s a virus. A virus the body deals with it and bacterial is when you use antibiotics.

P46 (About antibiotics) Doesn’t work for colds and flu.

P32: Half the time the doctors don’t prescribe antibiotics when they’ve got colds. Yes, it doesn’t even come up. They check them, they send them home with Panadol.

### Theme 2. Perceptions: factors that influence when and why to seek medical care for URTI

**Fig. 2:**
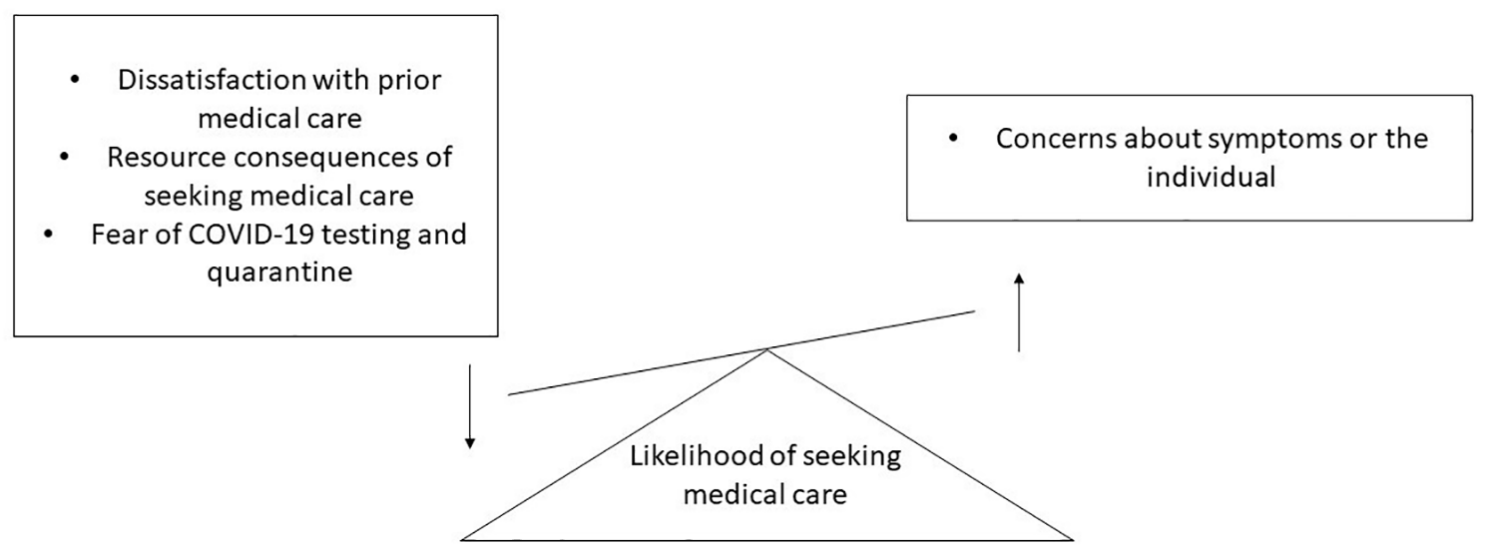
Theme 2: Perceptions - factors that increase the likelihood of seeking medical care and barriers that limit seeking medical care

Participants reported four subthemes that influenced their likelihood of seeking medical care for a URTI (Fig. 2).

(a) Concerns about symptoms or the individual.

Participants identified several issues that influenced their decisions to seek medical advice, and/or antibiotic treatment for their children or themselves. For parents, the decision to go to their GP was based on the severity of their child’s symptoms. These symptoms included fever, sore throat, listlessness, prolonged illness, and not eating or drinking. Participants also stated that they were more likely to seek medical advice if they or their children had chronic health conditions, wanting advice and reassurance. Participants who expected antibiotic treatment also stated that their expectations were related to the severity of the symptoms or that they or their child had a long-term health condition or that the person was elderly.

P17: Sometimes when there’s an infection you can physically see it or you can hear it in their chest if they have a cough. We just watch them be lifeless and lay there. Then I would expect to have antibiotics because they obviously can’t fight it.

P37: I’d take my kids anyway. But not only that, my kids have health issues. I don’t have a choice. Even if I go in and they send me out with nothing, at least I have peace of mind. I don’t want to wake up the next day and my kid is really sick.

(b) Dissatisfaction with prior medical care.

Some participants described feeling that a doctor had dismissed their concerns on a previous occasion. This feeling was often related to a failure by the doctor to acknowledge that the illness had persisted despite treatment with home remedies. Occasionally participants reported disappointment at not being prescribed antibiotics.

P15: When we go to the doctors, we expect them to give us antibiotics for our children. When they don’t prescribe it then, I don’t know. We’ve paid so much to see a doctor and we walk out and say we weren’t given anything; we were just given Panadol.

P17: So, when you’ve tried all that and then you’ve taken them to the doctor and then they’ve said to do the same thing but you’ve already done it for maybe a week, it comes down to are they actually listening? I’d say what I think. I’d say “I think that my son needs this, this is what is happening” and I’d just repeat myself.

(c) Resource consequences of seeking medical care.

Resource constraints commonly influenced decisions to seek medical care. These included the direct monetary costs related to the visit; transport costs and availability; needing taking time off work without pay; availability of sick leave; and waiting time. Some participants chose to use emergency care practices or a hospital emergency department in order to avoid the long wait times for an appointment at their usual general practices.

P30: Yes, even if you go in there and they tell you to come back at 6 o’clock, that’s another trip to town. You’ve got to pack the whole car up; you’ve got to put all the kids in and then you’ve got an hour waiting for the doctor to see you.

P2: Sometimes you go back three times. Come back, come back, come back. I think money is a big factor for Pacific people.

P36: Yes, you know your own child. If you see those six days later, they’re looking floppy of course you’re not going to leave them. Something’s wrong with them. It will not be to the doctors, it will be to the ED.

P43: What prevents me from going to the doctors is the time, the wait and lots of people there. It’s always packed. Not enough doctors on. That’s why it’s so full and there’s so many people there.

(d) Fear of COVID-19 testing and quarantine.

A few participants talked about the impact that COVID-19 had on their health seeking behaviour. Essential workers reported taking leave without pay when they were waiting for test results to see if their work colleagues had tested positive for COVID-19. Others discussed the requirement to have cold symptoms checked even though they weren’t concerned about the severity of the symptoms. Some participants talked about not wanting to report that neither they nor their children had URTI symptoms in case the children were required to get an unpleasant swab test.

P36: I think before COVID I used to take my kids all the time every time they had a snotty nose or something. Since COVID started it’s been a bit like “shall we go or look after them at home?”. Because you don’t want them to go through the whole testing. We’ve done a test and it’s not comfortable.

### Theme 3. Expectations: features of successful medical care for URTI

**Fig. 3:**
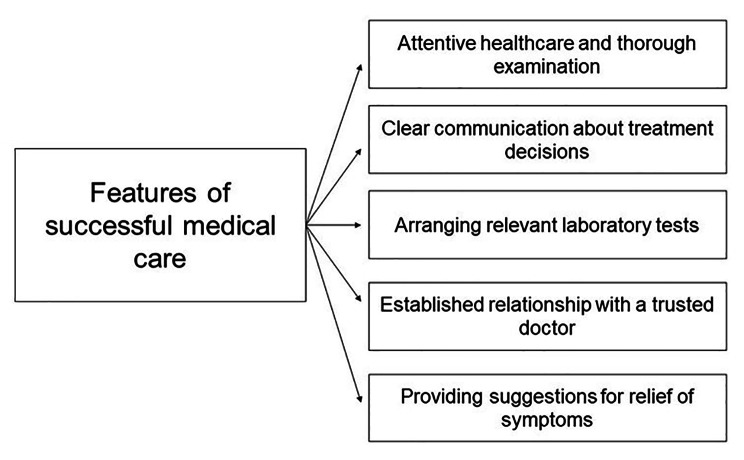
Theme 3: Expectations for successful medical care for URTI

Participants provided five subthemes describing the expectations of their ideal of successful medical care for themselves and their whānau (Fig. 3).

(a) Desire for attentive, high quality healthcare.

Participants expressed a desire to receive high quality healthcare. This included a thorough examination of themselves or their children; and for GPs to discuss relevant medical history.

P27:…at times when you go to the doctor, they see everything so often that you kind of feel like they’re never actually properly looking at what’s in front of them. So that takes away confidence in what they’re saying… that’s where the uneasiness comes in. You’re like, I don’t want to be a statistic, I want you to look at my kid.

P43: The whole thorough check, all over again. Checked the ears, the temperature, the thing in the mouth, checked the back of the throat, the breathing thing. Yes, held the tongue down to see if there was any inflammation or anything like that going on in there and therefore they could make their diagnosis from that and prescribe the medicine.

(b) Clear communications between patient and doctor about treatment decisions.

Participants highlighted the importance of having clear communications that they could understand, particularly when discussing different management options for their symptoms. Participants emphasised that information alone may not be enough, they wanted explanations about the rationale for treating (or not treating), without the use of jargon.

P9: An explanation is important. And they’ll always say to me, if we take our mother, they’ll say they’ll explain why we can’t do this and that. They’ll say is she all right with that, why? The reasons why. Which is really good.

P1: I think the good thing with our doctor is if they don’t give us antibiotics they’ll let us know the reason why. It’s a virus, it’s not going to help you.

P17: I think just breaking the words down and not using doctor terms but using normal people terms.

(c) Arranging relevant laboratory tests.

Parents living in areas where rheumatic fever is a major concern expected health care staff to routinely obtain swabs when their children had sore throats. In contrast, the participants in the Taranaki focus group, where rheumatic fever is much less common, were less concerned about the need for throat swabs.

P45: I’d ask aren’t you going to take a swab. I want you to take a swab. So you don’t have to come back again.

(d) Established relationship with a trusted doctor.

Participants wanted, if possible, to see the same GP on each visit to their doctor, and to build a good relationship with this person, as this helped them feel assured that all their concerns would be addressed.

P43: … like a small family doctor, I think they just actually care and make a connection. There’s a big one around the corner here where people are just coming and going and you’re just a number. You can feel whether they actually genuinely care or not.

(e) Suggestions for non-antibiotic relief of URTI symptoms.

Most participants were open to suggestions for alternative management of their symptoms and agreed that they wouldn’t have a problem with not being prescribed antibiotics, particularly if the decision was communicated effectively. They discussed the use of symptomatic medication and home remedies for treating their cold such as paracetamol, ibuprofen, lemon and honey, apple cider vinegar and increased water intake.

P14: I wouldn’t have a problem with it. I would ask them why. But I wouldn’t be disappointed or anything if they said no antibiotics needed because there’s other things available to you. Lemon and honey, and paracetamol, ibuprofen.

### Theme 4. Solutions – how to build community knowledge about URTI and their treatment and prevention

(a) Building community knowledge about antibiotics and URTI.

Participants recommended several solutions to build general public awareness that antibiotics are ineffective for colds. Participants identified a range of community settings such as marae (meeting places), churches, community centres, early childhood education centres, schools, sports organisations, and markets, where information could be shared and be accessible to communities and allow for discussion. Participants also suggested that multiple channels could be used to enhance engagement (e.g. Facebook, Tik Tok, radio and television). Participants agreed that print or written material alone would not be useful. Participants stated that any app developed to build knowledge would need to be engaging, and that acceptability would be increased by using a trusted member of the community as the spokesperson.

P1: I don’t think the pamphlets are going to work. It’ll be like I got this given from school but the last thing you want to do is sit there and read a pamphlet. You’re like “oh no”.

P7: … but actually having their own people explain it to them. I’m Samoan but I can go anywhere in the world, and I can meet another Samoan…I know them because we have a connection. It’s like anywhere you go, if I was to go somewhere to fill forms, if I had a Pacific person and you, I’d always go for the Pacific person.

(b) Effective communication about antibiotic prescribing decisions would help reduce expectations to receive antibiotics for URTIs.

Building on subthemes 3a and 3b, participants explained that some of the solutions that would assist them to understand antibiotics would be to receive more information on the specific action of the antibiotics along with the side effects and contraindication to other medications. A desire to be given clear information on the advantages and disadvantages of taking antibiotics and their effects was voiced by the participants. Participants stated that being given a clear explanation about why antibiotics do not work for colds would help them understand why antibiotics were not prescribed. Participants also stated that advice to stop taking antibiotics once you or your child starts feeling better (unless antibiotics have been prescribed for ‘strep’ throat) would be useful.

P1: I think the good thing with our doctor is if they don’t give us antibiotics, they’ll let us know the reason why. It’s a virus, it’s not going to help you.

P14(M): I guess information. Having the right information is key. Then if the doctor explains why and all that, and they generally do, then I make my own diagnosis sometimes and come up with the right solution.

## Discussion

This is the first large qualitative study to explore the knowledge, perceptions, expectations of both Māori and Pacific peoples in relation to antibiotic treatment for URTIs. The study participants highlighted several factors that are likely to influence their expectations to receive antibiotics for URTI. Limited knowledge about antibiotics and the illnesses for which antibiotics are beneficial are key drivers of a person’s expectations to receive antibiotics for viral URTIs. An individual’s perception of the severity and nature of the symptoms experienced also influences their decision-making about whether or not to expect antibiotics. While only a minority of participants knew that antibiotic treatment was not effective for most URTIs, the knowledge gained during the COVID-19 pandemic enabled some participants to recognize that antibiotics were not effective treatments for viral infections. Improving patients’ knowledge about the futility of antibiotic treatment for URTIs and alternative remedies to manage URTI symptoms may reduce their expectations for antibiotics.

Prior studies in NZ and overseas commonly have found similar gaps in knowledge and information. A recent qualitative study of Māori adults found that lack of knowledge and information, together with a person’s illness perceptions and treatment beliefs are key influencing factors on antibiotic expectations [12]. However, this study was conducted with participants from only one primary care practice in Auckland. A study of the knowledge and beliefs of 112 Samoan people resident in New Zealand during 2005–2006, found that 50% believed that antibiotics killed bacteria, 65% believed that antibiotics killed viruses, and 81% believed antibiotics were useful for URTI [20]. A nationally representative survey of 1509 Australian adults found that only 36% knew that antibiotics kill bacteria but are ineffective against viruses [21], and a survey of 1767 adults in England found that 24% believed that antibiotics work on most coughs and colds [22].

A small minority of our study participants stated that they would either insist on a prescription for an antibiotic, and if this was not provided, would promptly seek an alternative assessment by another doctor in the expectation of an antibiotic prescription. These findings are broadly similar to those of previous studies in New Zealand. A telephone survey of 400 randomly selected adults resident in Auckland, conducted in 1998 and repeated in 2003, found that only 20% had consulted a doctor about their most recent URTI. Approximately 50% of those who had ever consulted a doctor about an URTI did so to obtain an antibiotic [23]. In a prior study of adults and children with URTIs presenting to their GP, we found that approximately 40% expected antibiotic treatment [24]. In England, only 20% of survey respondents had consulted their GP about a recent URTI, but 50% of those who consulted their GP expected to be prescribed an antibiotic [22].

Although some of our study participants recalled prior dissatisfaction with not receiving antibiotics in situations when they considered these were required, dissatisfaction most commonly resulted from suboptimal communication of treatment decisions and rationale for these. These past experiences are likely to have reduced some participants’ willingness to return for medical care. Other factors that had a negative impact on returning for care were resource constraints – particularly related to monetary costs and time, including time off work and transport time.

A strength of using the Hui process and Talanoa method is that knowledge was shared and built during each focus group. At the end of each focus group participants enthusiastically provided suggestions to build community knowledge in multiple contexts and through multiple channels in order to reduce expectations about receiving antibiotics for colds. These suggestions primarily included the use of engaging informational material delivered in audio or video formats, delivered by a trustworthy source. Trust was an important consideration for communication to individuals and families about treatment decisions (e.g. from a trusted, known healthcare worker), and for general informational material (e.g. from a trusted spokesperson).

Our study has several limitations. We undertook a qualitative study approach which aimed to explore in an in-depth manner the views of Māori and Pacific adults. The study methodology was not intended to quantify the number of people who agreed with the themes that arose during discussions. Our findings might not reflect those of the entire New Zealand population – we specifically only recruited Māori and Pacific peoples to hear the voices of people facing the greatest health inequities, and only a few men (4/47) participated. Despite this, improvements that stem from the knowledge gained from our study participants will be of benefit to the entire population.

In summary, knowledge about URTIs has grown because of the COVID-19 pandemic, and this provides a platform to continue to grow knowledge. When people seek medical care for URTI, prescribers should consistently inform them that a virus is the likely cause, that antibiotics do not work against viruses, and that time and symptomatic treatments are all that is required for recovery. Many people seek treatment for their URTI and use of traditional and other home remedies should be understood, supported and encouraged by healthcare workers. While many people seeking medical care for URTI do so because they perceive the illness to be severe or are concerned that the illness could lead to serious illness, they are comfortable with not receiving antibiotics, provided a thorough assessment is performed and advice is clearly communicated. Many participants stressed that this advice should include describing the symptoms and signs that should lead to a repeat consultation. Finally, our focus group participants felt that reducing antibiotic treatment for URTIs is an important topic that warrants innovative user-friendly public information to reduce expectations to receive antibiotics, and ultimately lead to reduced unnecessary antibiotic consumption.

## Data Availability

(ADM) The datasets generated and/or analysed during the current study are not publicly available due to the private and confidential nature of the focus group recordings, but are available from the corresponding author on reasonable request.
